# A Synopsis of the Symptoms, Diagnosis, and Treatment of the More Common and Important Diseases of the Skin

**Published:** 1845-03

**Authors:** 


Art. VI.—*A Synopsis of the Symptoms, Diagnosis, and Treatment of the more common and important Diseases of the Skin.* With sixty colored figures. By N. Worcester, M.D., Professor of Physical Diagnosis and Pathology in the Medical School of Cleveland, late Professor in the Medical College of Ohio. Philadelphia: Thomas Cowperthwait & Co. Boston, Charles C. Little and James Brown. Cincinnati, Desilver & Burr. 1845. pp. 292. 8vo.

We must agree with Dr. Worcester that a compendium of the kind here presented to us has long been needed. We are disposed also to admit the truth of what he says concerning the little attention that cutaneous diseases have received at the hands of the general practitioner. They seem to have been shunned both by physician and surgeon, and to have been delivered over, by general consent, to quacks, and to old women, whether in petticoats or cut-a-way coats. These last have found their peculiar home in this department. Here they have revelled, rioted, luxuriated; here they have reaped their greatest harvest. Hence the legion of popular nostrums whose virtues we hear vaunted, from *Judkin's Patent Specific* up to that of — we forget the name—with Duff Green's recommendation. Every old granny, whether male or female, has her "nice sa've," her "'intment," not forgetting the "persipity," white and red; and her *teas* from "sassyfrac," or "pennyr'yal," down to "sheep-saffron," to purify the blood. We have not the difficulty our author has in accounting for this. None deny the importance of these diseases, their frequent occurrence, and the facility of examining them. It may be, too, that they are susceptible of a "simple, beautiful, and perfect classification;" but, nevertheless, it is the absence of this, we apprehend, that constitutes one of the causes of their neglect. Students have stopped, appalled by the inextricable confusion of tongues that greeted them at the threshold, and perplexed by the multiplied and excessive refinements that met them at the outset. The desire of presenting something new, has been attended with as unfortunate results here as elsewhere. Authors seem to have puzzled their brains, and certainly they have perplexed those of others, in their hair-splitting distinctions and divisions. Alibert's tree is but a type of them. One of the latest works on the subject makes no less than nine varieties of erysipelas! Names are so accumulated, so piled and heaped on each other, so *imbricate*, to use a botanical phrase, as to form a covering as impenetrable as the bull's-hide shield of the Roman soldier, or the seven-fold one of Ajax. Is it any wonder that these diseases have been neglected? that a physician in laborious practice has turned from them almost in disgust? Another reason, perhaps, for this neglect, is the expensive nature of most of the special treatises—they are not within the reach of the general practitioner. And a third reason is the inadequacy of mere description, however accurate, to fix their characters in the mind, and the difficulties of seeing examples in a sparse country practice.

Dr. Worcester does not pretend to any originality in his synopsis, but candidly tells us that he has drawn his materials from every source that he found available. In the classification of cutaneous diseases he has followed Willan, not because the system of the latter is without strong objections, but because, notwithstanding its defects, it is the "most simple," and "affords greater facilities for detecting and classifying a disease." Willan's arrangement seems to bear about the same relation to that of other authors that Linnæus's artificial system of botany does to that of more recent writers on the same subject—less scientific, or philosophical, but absolutely indispensable to the student or young beginner. It has been called, in fact, a work on natural history rather than pathology. The new orders that have been added to the system by more recent writers, consisting, for the most part, of a single disease, and this of rare occurrence, have been omitted by our author, as not coming within the scope of a work designed as a manual of the more common diseases only. He has also omitted the eruptive fevers, as a minute account of them is contained in most of the treatises on medicine. To facilitate diagnosis, Dr. W. divides the orders into two groups, the *moist* and the *dry*. In the first group we have three orders, in the second five. Thus:

*First Group.*

| | |
|---|---|
| VESICULÆ. | Eczema. Herpes. Scabies. Miliaria. |
| BULLÆ. | Pemphigus. Rupia. |
| PUSTULÆ. | Ecthyma. Impetigo. Acne. Mentagra. |

*Second Group.*

| | |
|---|---|
| EXANTHEMATA. | Erythema. Roseola. Urticaria. |
| PAPULÆ. | Lichen. Prurigo. |
| SQUAMÆ. | Lepra. Psoriasis. Pityriasis. Ichthyosis. |
| TUBERCULÆ. | Elephantiasis. Molluscum. Frambesia. |
| MACULÆ. | Ephelis. Nævi. Albinismus. Vitiligo. |

He gives the following simple rules in making a diagnosis:

"1st. Ascertain whether the eruption was originally dry or moist

"2d. Examine with great care the eruption, and in a great majority of cases some examples of the elementary form of the eruption, as the papule, vesicle, or pustule, may be found either in the neighborhood of the part principally affected, or in some more recent eruptions. Almost all cutaneous diseases are successive in their appearance, which is often of great assistance in the diagnosis.

"3d. Where you cannot satisfy yourself by a careful examination, as to the elementary form of the eruption, after having ascertained the group to which it belongs, try to learn from the patient or friends the appearance of the disease at first, as whether the vesicles were from the very first filled with a clear, limpid, or with an opake and yellow fluid; whether the pimples were at first distinct, small, hard, and conical, or were flattened, soft, conglomerated, &c. &c.

"In most cases by observing these three general rules, not only the group, but the order to which the disease in question belongs, can be ascertained; beyond this the differential diagnosis of the different diseases belonging to the same order, is commonly not difficult and often of no great practical importance."

The prognosis is not unfavorable if these diseases are treated in the early stage. It is only when they are allowed to continue until the delicate structure in which they are seated is disorganized, that they become inveterate.

In relation to the causes of cutaneous affections, our author presents nothing new. Some are hereditary, some contagious, some are excited or kept up by certain articles of food, but in many instances no satisfactory cause can be ascertained. Dr. W. thinks the most prolific source in this country is want of cleanliness; the same cause was assigned by Willan for their frequency in England.

On the subject of treatment Dr. W. insists that if the true pathology is kept steadily in view, and remedies are applied according to the character and stage of the disease, the result will be as satisfactory as in the diseases of other organs.

"The great reason that the treatment of cutaneous diseases has generally been attended with so little success, is that we prescribe for them too empirically, and do not pay sufficient attention either to the pathology or to the stage of the eruption. The same general principles should govern us here, as in the treatment of all other diseases; a great proportion of cutaneous eruptions are at first inflammatory; in this stage, common sense would direct general depletion by blood-letting and cathartics, and local soothing applications, as cataplasms, water-dressings, &c., to allay the local excitement; in short, a strict, antiphlogistic course, both in medicine and regimen, should be directed. When the acute stage has passed by, the course should then be changed, and in some cases the treatment can now be beneficially reversed, and tonics or even stimulants, both general and local, be substituted for depletion."

Many of these affections are maintained by improper treatment long after they would otherwise have ceased. The practitioner should also bear in mind the vicarious office of some of them both in the adult and the child; and should be cautious how he interferes with them before attending to the general health. Cleanliness is of vast importance, especially in diseases belonging to our author's first group; nothing else being requisite for the cure of many of them. Attention to diet is also of great importance. Scanty and innutritious food is known to excite cutaneous diseases; hence among the poor and destitute of large cities, a generous diet is about all that is required. "A mild, unstimulating, abstemious diet in the acute stage, followed by a more generous allowance, as the activity of the symptoms abate, is found to be the general rule in the treatment of all these diseases." Certain articles of food have, from time immemorial, been set down among the exciting causes of cutaneous eruptions. The Jewish prejudice to pork seems to be well founded. Salted meats, articles of a fatty or oily nature, heating condiments and spices, and alcoholic drinks, are to be avoided.

Among the general remedies, the lancet and purgatives are peculiarly useful in the active stage of most eruptions; next to these come tonics, especially the bitter infusions and mineral acids. Advantage is frequently found in giving the saline cathartics in bitter infusion, or with a few drops of one of the mineral acids. The great remedy at the present day is arsenic, in the form of Fowler's or Pearson's solution, the Asiatic pill, or combined with iodine and mercury as in the triple compound recently introduced under the name of Donovan's solution. Cantharides in the form of tincture, and corrosive sublimate, are valuable in the same cases and stages as arsenic, with which they are frequently alternated. Sulphur in substance, or combined, as in some of the mineral waters, is another remedy of considerable efficacy. The dose should not be large, and it should never act as a cathartic. Two new remedies have lately been introduced, the anthrakokali and fuligokali. Our author gives the formulæ for their preparation, but does not seem to place much confidence in them. Of local remedies, leeching and sedatives are most important in the active stage of eruptions; in the chronic stages, stimulating applications are required. Among the soothing remedies, the author, as might be inferred from what we have already stated, places foremost some variety of the general or local warm bath. Of the stimulating applications, none are so generally useful as some of the sulphur baths. They are a favorite remedy with Alibert, and at the Hospital of St. Louis, in Paris. They are found most useful in the same cases as arsenic, that is to say, where the eruption has become squamous.

Such, in a general way, are the principles which, according to our author, should guide the practitioner in the treatment of cutaneous affections, and such are some of the means of treatment. We cannot, of course, follow him in his description of the various orders and genera of diseases. These descriptions are concise, but accurate; while, under the several heads of *diagnosis*, *prognosis*, *causes*, *and treatment*, will be found all that, in the present state of the science, is known of each particular disease.

The mechanical execution of the work must not pass unnoticed. Judgment and taste have been evinced in selecting a large, clear type, with a page not too much crowded—two things which, in our opinion, are no mean recommendation. Among the colored figures, which are absolutely indispensable in a work of this kind, will be found an illustration of nearly every disease mentioned in the text. These are executed in a style creditable to Western art, and are, so far as we can determine, faithful to nature.

In regard to the literary merits of the volume, we might, with good reason, take the author severely to task on account of numerous ill-constructed sentences, and a frequent looseness and carelessness of phraseology. But he is young as an author, and we are disposed to let these pass without comment. We are not sure, however, that the publishers do not deserve a rap over the knuckles for the many, and, in some cases, monstrous errors of the press. These are so many blemishes on the work, which might have been and should have been guarded against. C.

